# South Korea’s Health Misinformation Response during COVID-19: A Narrative-Thematic Analysis

**DOI:** 10.1007/s41649-024-00323-3

**Published:** 2025-06-16

**Authors:** Sophia Wasti, Hajeong Lee, Hannah Kim

**Affiliations:** 1 Asian Institute of Bioethics and Health Law, Yonsei University, Seoul, Republic of Korea; 2 Division of Medical Humanities and Social Science, Yonsei University College of Medicine, Seoul, Republic of Korea

**Keywords:** Health misinformation, Infodemic, COVID-19, Vaccine skepticism, Social media, South Korea public health policy

## Abstract

Infodemics have emerged as a serious contemporary challenge to public health, especially in the context of the ongoing COVID-19 pandemic. This paper conducts a narrative thematic analysis exploring the South Korean response to the public health risks caused by misinformation, critically examining the legal, social, and ethical dimensions of dealing with the difficulties posed by health misinformation, identifying the following key themes: limitations posed by existing law in South Korea, government policies as a response to the COVID-19 pandemic, self-regulation in the private sector and mitigation of the social impacts of COVID-19 misinformation. The paper offers a thematic exploration of South Korea’s integrated policy response to health misinformation within the context of the global COVID-19 infodemic and highlights the South Korean effort to balance the protection of public health and welfare with citizen’s individual rights to freedom of expression and the necessity of flexibility and adaptive policies to effectively counter COVID-19 misinformation. It observes the importance of effective public health communication and provides insight useful for dealing with potential future challenges arising from the proliferation of health misinformation and mitigating the adverse impacts of infodemics on public health initiatives, using the example of South Korea.

## Introduction

The COVID-19 pandemic has subjected global public health systems to serious challenges as the foremost global health crisis in recent history. It is the first pandemic of its kind to affect and alter life on a global scale as high rates of person-to-person spread combined with comparatively high rates of mortality led to the declaration of an international public health emergency by the World Health Organization (WHO 2020a). In response, nations across the globe adopted various measures, differing in scope and approach, aiming to stem the spread and mitigate the impacts of the virus. During the pandemic’s initial stages, especially, the general public turned to online spaces as a source for healthcare information related to COVID-19 (Barua et al. 2020). Content spaces online content equally accommodate accurate and inaccurate health-related content, with the potential to circulate misleading advice, unfounded claims, and unverified remedies, exposing individuals to the risks posed by health misinformation. Factors such as sensationalism, confirmation bias, and the echo chamber effect are some factors speculated to contribute to the rapid proliferation of health misinformation (Flintham et al. 2018). This paper considers the response of South Korea in the context of the problems presented by the infodemic associated with COVID-19, exploring social, legal, and ethical dimensions of the issues associated with managing health misinformation using a thematic analysis framework against the background of the general response to the challenges posed by the COVID-19 pandemic. The purpose of this paper is to understand the nature of South Korea’s policy interventions and legal measures and the extent to which they aimed to address the challenges of health misinformation during the COVID-19 pandemic via the lens of its general response measures.

In light of the perceived general success of preventive measures and initiatives designed to mitigate the spread of COVID-19 (Kim et al. 2021), this paper looks to highlight the South Korean government’s approaches to public engagement and communication as a key component of government strategy in tackling the issue of health misinformation (Yoo et al. 2021) especially within the context of preserving the values associated with the fundamental right to freedom of expression enshrined in Article 21 of the Constitution of the Republic of Korea (1987). The period of analysis spans the initial emergence of the virus in January 2020 through to May 2022, when pandemic-related restrictions were beginning to ease (Lim and Sohn 2023). Misinformation concerning COVID-19 was recognized as critical to promoting preventive behaviors (Lee et al. 2021b) during which the government collaborated with various sectors in order to implement a range of measures in order to curb the spread of the virus and counteract the proliferation of misinformation (Oh 2020).

## Methodology

The paper employs an adapted version of Braun and Clarke’s (2006) approach to thematic analysis using an inductive approach to explore the various ethical, legal, and social dimensions of South Korea’s response to the COVID-19 infodemic. The coding process was omitted in order to facilitate a more dynamic approach to the interpretation of the themes, as the sources used for analysis spanned a wide range of types of literature, from legislation and policy documents to press releases and various studies. This analysis involved a comprehensive review of a variety of data sources, including governmental policy documents, legislation and legal case analysis, public health communications, and media reports. The themes derived were used to develop a comprehensive perspective on the South Korean policy response to the infodemic.

- Initial literature review: Various sources, including legislation, legal cases, government policy documentation, private sector initiatives, media reports, and scholarship about South Korea’s actions during the COVID-19 pandemic were collected and analyzed by members of the research team.
- Theme definition: Themes were identified directly from the literature focusing primarily on the legal and policy responses, ethical considerations, and societal impact, defined during the course of the review. Themes were later refined for specificity throughout the analysis stage, making sure they accurately reflected the spectrum of South Korea’s responses to the infodemic. The following themes were identified during the course of the review: limitations posed by existing law in South Korea, government policies as a response to the COVID-19 pandemic, self-regulation in the private sector, and the mitigation of social impacts of COVID-19 misinformation.
- Analysis: Themes were analyzed with special consideration given to the research team’s perspectives and interpretations of the issues, shaping understanding of the emerging themes.
- Final review: The information was reviewed for consistency and accuracy.

## Background

### Implications of Infodemics for Public Health Messaging

The sheer amount of information available concerning COVID-19 during the initial stages of the outbreak was being published to the internet at an incredible volume and rate (Škorić et al. 2020), including information that may have been false, misleading, or outdated. This has contributed to a massive scale infodemic unlike any involved with prior global health events. Compared to traditional media or in-person interactions, the comparative simplicity of generating and distributing news on the internet leads to the production of vast amounts of fake news for various purposes (Shu et al. 2017). The term ‘infodemic’, refers to the ‘overabundance of information, both online and offline’… and ‘includes deliberate attempts to disseminate wrong information to undermine the public health response and advance alternative agendas of groups or individuals’ (WHO 2020b). Equally, the proliferation of so-called fake news associated with COVID-19, characterized by the dissemination of false or misleading information presented as legitimate news content, has potentially profound implications for public health messaging, where that messaging can be undermined by political or social agenda (Egelhofer and Lecheler 2019). In recent decades, the term ‘fake news’ has been strategically employed to delegitimize messaging and credibility and cast doubt on the reliability of official sources. A source’s association with the term may influence the perception and categorization of news content as fake or real, affecting whether the public chooses to accept its messaging (Baptista and Gradim 2022). The World Health Assembly (2020) operating within the remit of the World Health Organization took action to recognize the potential impact of the spread of misinformation in the Resolution WHA 73.1, and managing misinformation is a critical part of controlling the spread of COVID-19. However, legislation and measures to effectively manage the spread of misinformation have been slow to develop in response (Madhav et al. 2017). The apprehension and subsequent delay stem from the complexity of addressing this issue within a public health context, where efforts to combat misinformation can clash and must be balanced against the fundamental right to freedom of expression. Regulating health misinformation is especially contentious owing to the intersection between media and public health initiatives and information and parallel methods of communication. Additionally, the imposition of limits on personal liberties and freedoms of expression has become a key focus area of legal and ethical deliberation throughout the course of the COVID-19 pandemic and the various efforts made to contain the spread of the infection. These problems are pervasive, adversely affecting many aspects of life for people all over the world, but as governments and policymakers grapple with the difficulties of responding to the problem of health misinformation there are opportunities to examine how these tensions manifest within specific national contexts. Despite growing recognition that public health misinformation can act as a barrier to implementing successful health initiatives, there remains a lack of concrete data illustrating a direct cause-and-effect relationship between exposure to misinformation and its influence on individual health-related decisions (Greene and Murphy 2021). Effects of different types of content, the dynamics and psychological drivers of misinformation sharing are all areas where information is lacking on the likely impact on individual health or behaviors (Chou et al. 2020) and the effects on different communities at the public health scale. The Organisation for Economic Co-operation and Development (OECD 2023) has issued advice based on 9 key principles for effective public messaging and communication: (1) transparency, (2) inclusiveness, (3) whole-of-society collaboration, (4) public interest-driven, (5) institutionalization, (6) evidence-based, (7) timeliness, (8) prevention, (9) future-proofing and professionalization emphasizing the need for a multi-disciplinary, multi-stakeholder approach to public communication.

### Overview of Measures During the COVID-19 Pandemic in South Korea

In early 2020, the South Korean government implemented rapid and preventive measures to help combat the spread of COVID-19. These measures included the tracing of potential infection routes and enforcing strict isolation and quarantine protocols for confirmed patients (Lim and Sohn 2023). High-risk groups, particularly those with underlying health conditions and the elderly, were strongly advised to wear N95 or KF94 masks, with mask mandates in place for all public indoor spaces, including transportation. Fines were imposed for non-compliance (Lim and Sohn 2023).

Diagnostic testing was, equally, a central component of the strategy, with immediate testing for individuals displaying symptoms and those entering South Korea from abroad. Depending on the severity of COVID-19 symptoms, individuals were either directed to self-quarantine or hospitalized (Lim and Sohn 2023). Coupled with the voluntary cooperation of the general public, these measures yielded comparatively more favorable public safety outcomes, resulting in far lower mortality rates than in areas such as the USA, UK, and Europe as a consequence (Johns Hopkins Coronavirus Resource Center 2023). The measures implemented effectively reduced the impact of COVID-19 in South Korea, but difficulties associated with tackling vaccine misinformation, evidenced by the significant vaccine refusal rates and the spread of negative information on social media, indicate the complexity involved in developing public health policies that effectively counter misinformation. In May 2021, South Korea secured supplies of both the AstraZeneca (AZ) and Pfizer vaccines and undertook a vaccination campaign targeting healthcare professionals and individuals aged 60 and over (Lee et al. 2021a)*, w*ith approximately 2% of the overall population receiving one dose of the vaccination with goals to inoculate a minimum of 70% of the population by December 2021, aiming to attain a level of herd immunity (Lee et al. 2021a). Several studies have highlighted the importance of widespread vaccination acceptance as a prerequisite for attaining herd immunity (Frederiksen et al. 2020), however according to a study undertaken by Lee et al. (2021a) the vaccine refusal rate stood at 33% among the South Korean public, with many individuals opting out of receiving the COVID-19 vaccination despite being eligible (Lee et al. 2021a). The proliferation of negative information about the vaccines on social media fueled hesitancy and skepticism towards the COVID-19 vaccination in South Korea where negative perceptions associated with the AZ and Pfizer vaccines informed a level of skepticism and hesitancy towards the vaccination in general (Lee et al. 2021a). Consequently, there was concern from experts in the field of vaccination that South Korea would be unable to achieve herd immunity as the percentage of vaccinated individuals did not meet the prerequisite 70% potentially compromising the country’s vaccination strategy (Lee et al. 2021a). However, the late-stage plateau of the vaccination rate in South Korea after surpassing the 80% mark (Choi et al. 2022), (attributed to a subset of the population opposing vaccination (Choi et al. 2022), indicates a generally high level of trust in the Korea Centers for Disease Control and Prevention (KCDC) among South Koreans. Trust in government agencies responsible for developing policies related to disease prevention and control was significant and suggests that a higher level of trust in government made South Koreans less susceptible to conspiracy theories and misinformation about vaccines (Choi et al. 2022). Additionally, efforts to mitigate the impact of misinformation and disinformation on vaccinations, such as deleting fake news on social media platforms, highlight the importance of a collaborative approach in countering false information to maintain public trust in healthcare institutions highlighting the impact of trust in public institutions on public health outcomes. Conversely, lower trust in government has been found to be inherently relevant to vaccine hesitancy, indicating that government communication and initiatives can impact vaccine acceptance (Lee and You 2022). Additionally, the use of offline and online media as sources for perceived benefits of vaccination was associated with vaccine hesitancy, suggesting that effective use of media is key to public health messaging and shaping perceptions that can potentially affect vaccine uptake (Lee and You 2022). Similarly, trust in public health messaging and institutions is linked with health literacy levels, influencing vaccine acceptance rates and the ability to combat misinformation effectively. Enhancing health literacy can bolster public trust and facilitate informed decision-making regarding COVID-19 vaccination (Song and Lee 2023). Health literacy is equally significant. Levels of health literacy have been found to influence attitudes towards vaccination in South Korea (Song and Lee 2023). Concerns about vaccine safety were more apparent among individuals with lower health literacy levels (Song and Lee 2023). Individuals who encounter or experience difficulty in accessing and understanding health information are at a higher risk of vaccine hesitancy compared to those with proficient health literacy skills. The acceptance of COVID-19 vaccines is closely linked to the capacity to discern and identify ‘fake news’ resources and health literacy levels. Individuals with higher health literacy are more likely to critically evaluate information and make informed decisions regarding vaccination (Song and Lee 2023).

### Government Strategies Responding to Mis-/Disinformation in South Korea

The governmental response to misinformation during the COVID-19 pandemic in South Korea integrated legal measures, policy interventions, and public engagement strategies. The Korea Internet Self-Governance Organization (KISO) has established policies and regulations to address the distribution and removal of specific types of content (KISO 2018), including guidelines covering content specifically related to COVID-19 in Article 34 of the KISO Policy on False and Manipulated Information Regarding COVID-19 (2021). The scope of content subject to removal is exclusively concerned with COVID-19-related matters, such as information on its existence, treatment, prevention, diagnosis, infection, social distancing, and self-isolation. Limiting the categories under investigation specifically to ongoing issues serves to prevent overreach and ensures that removal actions are targeted and relevant. This includes content that impersonates or falsely uses the identity of media organizations or content with false information (KISO 2018). Specific policies relating to misinformation concerning COVID-19 were issued in 2021 (KISO 2021b), aiming to address the problems associated with false information while preserving the value and importance of freedom of expression. These policies, introduced in April 2021 and concluded in March 2023, specifically concerned the distribution of COVID-19-related information (KISO 2021b). The Korea Communications Commission (KCC) also introduced comprehensive measures to combat COVID-19 vaccine-related fake news (KCC 2021b), establishing a Vaccine Disinformation Reporting Board to address issues related to vaccine misinformation (KCC 2021a).

The Korea Communications Standards Commission (KCSC) holds authority for post-review evaluations of broadcast programs and advertisements by broadcasting companies under current law. They can take various actions against illegal harmful information distributed on the internet, such as content removal, user access suspension or termination, and ensuring compliance with displaying harmful information to minors (Enforcement Decree of the Act On the Establishment and Operation of Korea Communications Commission 2018). There has been a collaborative effort between agencies to manage mis-/disinformation related to COVID-19 involving a ‘Promotion and Fake News Response Council’, which includes participation from six different government agencies: The Korea Communications Commission (KCC), Ministry of Health and Welfare, Ministry of Culture, Sports and Tourism, Korea Disease Control and Prevention Agency (KDCA), Korea Food and Drug Administration (KFDA), and the Korean National Police Agency (KNPA) (Ministry of Health and Welfare 2021). Their aim is to create digital content such as card news (card-based news articles) and short video clips to address and combat misinformation surrounding COVID-19 vaccination (Ministry of Health and Welfare 2021). In 2020, the Broadcasting Commission reviewed and took corrective actions on 4624 pieces of information related to COVID-19-induced social confusion, including issuing corrective measures for 200 cases (Hwang 2021).

### Private Sector Response to COVID-19 Misinformation in South Korea

Companies in the private sector also implemented self-regulatory measures in order to contribute to the efforts being made to combat health misinformation. Domestic web-portal sites, such as NAVER and Kakao, took steps to ensure the dissemination of accurate COVID-19 information aligned with government announcements (NAVER Corp 2021; Kakao Corp 2021). Application-driven data sharing emerged as another vital component of direct communication with the public, with developers creating tools to deliver up-to-date information about mask inventory, helping facilitate national inventory management and enabling individuals to access the information necessary for securing personal supplies (Task Force for Tackling COVID-19,2020). Domestic web platforms also made maps of screening centers and real-time updates on mask supply available to the general public to help keep them informed about availability of resources. Additionally, prominent media associations, including the Journalists Association of Korea (JAK), the Korean Broadcasting Journalist Association (KBJA), and the Korea Science Journalists Association (KSJA), introduced Epidemic Reporting Guidelines in 2020 (Journalists Association of Korea 2020). The guidelines served as a call for journalists to distinguish medical facts from opinions and refrain from using sensational language that could potentially incite social unrest. Other platforms with wider global reach and strong presences in South Korea like Facebook, Youtube, Twitter, and Instagram have also taken measures to combat misinformation and have implemented policies aimed at enhancing user awareness relating to the reliability and risk level of information (Benecke and DeYoung 2019) and actively supported fact-checking activities, using the results of these assessments to regulate content. COVID-19 saw these efforts intensified, and various features highlighting the trustworthiness, timeliness, and integrity of posts and content on their various platforms were introduced (Nunziato 2022).

### Dissemination of Misinformation

Internet media and traditional news reporting often prioritize novel information and anomalies (Pinker 2018, as cited by Belluz 2019), tending to material that generates more attention and be shared between different people and groups, especially older members of the population (Zhou et al. 2023). This makes more shocking, surprising, or sensational information preferable for these platforms from a promotional perspective. Social media platforms are also able to facilitate the rapid dissemination of mis-/disinformation and conspiracy theories related to COVID-19 (Dow et al. 2021). Panic and misinformation have the potential to spread more quickly than the time required for fact-checking, investigation, and corrections (Chou et al. 2020). This proves challenging for policymakers as there is evidence that the relationship between individual emotions, cognitive biases, and psychological factors potentially makes direct attempts to counter misinformation less effective and more difficult due to the compounded effects of fear and mistrust (Chou et al. 2020).

This phenomenon potentially presents substantial difficulties for public health messaging and management because of the speed with which it can spread across communities. It has the potential to undermine faith and erode trust in governments, healthcare systems and professionals, and medical treatment which has the potential to slow recovery and may put lives at risk (Silver et al. 2022). The challenges associated with managing the spread and impact of health misinformation are thought to have triggered resurgences of diseases that were previously on the decline or considered under control due to vaccination programs and public health infection control measures. In 2019, prior to the COVID-19 pandemic, measles outbreaks had reached emergency levels in multiple countries (Benecke and DeYoung 2019) as anti-vaccination sentiment has flourished in various online spaces (Benecke and DeYoung 2019).

Moreover, more immediate responses from social media companies, public health agencies, and regulators are often reactive and can only take effect once misinformation has already been widely circulated (Matasick et al. 2020). Additionally, social media companies have pushed back against the idea that they bear any legal responsibility for what gets posted to their platforms as information is uploaded and spread by its users (Helberger et al. 2018). Only in the years immediately prior to (2016 onward) and during the pandemic that these platforms have intervened in new ways to de-platform individuals responsible for spreading misinformation or blocking and highlighting posts containing misinformation (Chachko 2021). Evidence also indicates that belief in conspiracy theories can deter individuals from receiving vaccinations or necessary antibiotic treatments, further exacerbating public health challenges (Benecke and DeYoung 2019). This becomes significantly more challenging where conspiracy theories become intertwined with credible sources, implying the existence of concealed truths. Paradoxically, attempts to debunk such theories can inadvertently strengthen conspiratorial beliefs, fostering an ‘us vs. them’ mentality and facilitating even greater distrust (Agley and Xiao 2021).

### Approaches to Managing Misinformation

Regulatory challenges materialize in cases when efforts are being made to deal with uncertain information, particularly when it lacks validation from authoritative bodies such as the World Health Organization (WHO) or other reputable disease control agencies (Hwang 2021). The process has the potential to become particularly complex where efforts are being made to reflect changing official standards in line with domestic law and international guidance. Determining appropriate regulatory actions becomes more difficult, leaving gaps in regulation leading to ambiguity and uncertainty. When trusted authorities like the WHO alter their decisions or concerns emerge about the reliability of certain pieces of information, reinstating previously removed content becomes complex.

For example, early in the COVID-19 outbreak Facebook initially identified the claim that COVID-19 originated in a lab as false, applying strict penalties for sharing such misinformation. However, subsequent shifts in official stances, including acknowledgment of the theory’s plausibility by U.S. intelligence (Looi 2023), prompted Facebook to reconsider and remove the claim from its misinformation list (Hwang 2021). Similarly, KISO’s current standards also lack provisions for addressing the restoration of content after removal, further complicating content management.

During public health disasters like the COVID-19 pandemic, where there is often high informational uncertainty and limited predictability in scientific activity, there is usually insufficient evidence for media and regulatory bodies to make well-informed judgments. Consequently, legal, academic, and political debate often follow these official determinations, underlining the fluid and evolving nature of information during an emerging public health crisis.

A further aspect may involve the cultivation of informed citizens who are able to actively contribute to campaigns against health misinformation and generate reliable and correct information. Effectively countering misinformation involves the ability to critically assess the accuracy and credibility of various sources and articles. This may take the form of communication and informational campaigns encouraging understanding of media and digital literacy, not dissimilar to the Stop the Spread Campaign rolled out on BBC World television, the website, and its apps during May and June 2020, (WHO 2021c) encouraging people to check information with trusted sources such as the World Health Organization and national health authorities, therefore limiting the spread of false information by encouraging fact checking behaviors using trusted sources and authorities on information (WHO 2021c). An additional issue with these approaches to stemming the impact of health misinformation is that strategies are often only effective on English or native language versions of the platforms—there has been widespread and high-profile criticism of the lack of policies and initiatives concerned with tackling medical misinformation in other languages (Burke 2020) as this has the potential alienate non-native resident communities like expatriates and immigrant populations. Returning the human and familiar aspect to communication using narratives has also indicated proven effectiveness (Iles et al. 2022) during the COVID-19 pandemic and it may be worth looking at the successes of strategies in other countries that may be adapted and reapplied effectively elsewhere (Nsoesie 2021).

## Analysis

In the course of examining the literature on South Korea’s response to managing health misinformation, several distinct themes emerged.

### Limitations posed by Existing Law in South Korea

South Korea has consistently made efforts to implement legislative measures aimed at regulating online communications and the dissemination of false information through the internet and social media platforms recognizing the impact of telecommunications on the spread and visibility of information. Challenges have emerged in relation to key provisions of the Acts, specifically Article 53(1) of the Telecommunications Business Act in 99Hun-Ma480, highlighting the inherent tension between individual freedom of expression and protection of the public. On June 15, 1999, the complainant, a student from Hankook Aviation University, posted a message titled ‘Exchange of Gunfire in the West Sea’, ‘Sloppy Kim Dae-Jung!’ on the ‘urgent message board’ of the internet community ‘Chanwoomul’, managed Nownuri, a comprehensive computer network service provided by Nowcom, Inc. On June 21, a Nownuri system manager deleted the post and suspended the student’s service access for a month, acting under directives from the Minister of Information and Communication. On August 11, 1999, the student filed a constitutional complaint against the relevant provisions of the Telecommunications Business Act (specifically Article 53 and parts of Article 71(vii) related to Article 53(3)) and Article 16 of the Enforcement Decree of the Telecommunications Business Act. The student alleged that these provisions infringed on his freedom of expression as well as freedom of science and arts, that the provisions went against due process, and violated the principle against excessive restriction (Global Freedom of Expression n.d.).

The Court’s review was specifically concerned with the constitutionality of Article 53 and parts of Article 71(vii) concerning Article 53(3) of the Telecommunications Business Act, and Article 16 of the associated Enforcement Decree. These provisions govern how telecommunications services are managed and regulate how service providers may control content and user access in response to directives from the Minister of Information and Communication (Constitutional Court of Korea 2002a).

The majority consensus was that a prohibition on communication that could ‘harm the public peace and order or social morals and good customs’ lacked the necessary clarity to be considered legally effective (99Hun-Ma480 2002). The terms ‘public peace and order’ and ‘social morals and good customs’ used in the statutory provisions were held by the majority to be abstract and subjective and did not constitute appropriate guidelines for administrative regulation (Ban on Improper Communication on the Internet Case, 14–1 KCCR 616, 99Hun-Ma480 (Constitutional Court of Korea, 27 June 2002)). However, in their dissenting opinion, Justices, Ha Kyung-chull, Kim Young-il, and Song In-jun, held a different view from the majority on the constitutionality of Article 53(1) and 53(2) of the Telecommunications Business Act, disagreeing with the majority assessment that the relevant provisions were in violation of the Constitution. They maintained that Articles 53(1) and 53(2) did not infringe upon constitutional principles. They contended that these provisions, outlining the criteria for regulating improper communication, do not need to provide explicit definitions of what constitutes improper communication. Instead, they held the view that the task of specifying these details should fall to a presidential decree.

This perspective extended to the evaluation of Article 53(3) of the Act and Article 16 of the Enforcement Decree. They argued that the provisions under scrutiny, responsible for establishing criteria for improper communication that may be regulated, do not need to explicitly define the parameters of the communication, similarly delegating the responsibility of specifying these details to a presidential decree. In their view, the delegation of rule-making authority did not amount to an unconstitutional restriction on freedom of expression, contrary to the majority interpretation.

In terms of regulating the spread of misinformation, the dissenting Justices held the view that regulation is preferable to the absence of any legal framework despite the potential for ambiguity. The approach of employing the enforcement decree to delineate the specifics of improper communication introduces a more tailored approach to oversight. By assigning the responsibility of establishing precise criteria to a decree, the law may be tailored to the specific contexts of misinformation. However, following the reasoning upheld by the majority, shifting the responsibility to define key terms to a decree, the risk is that the criteria for what constitutes improper communication may be subject to the discretion of the executive branch without clear legislative guidance.

This may result in inconsistent application of the law, potentially ineffective limitations for administrative agencies responsible for delegated legislation, or potential abuse of power.

The Court expressed concerns that the ambiguity and comprehensiveness of these concepts could result in the regulation of communication that should not be restricted, potentially violating the rule against excessive restriction. The majority decision highlighted the risk of regulating protected expressions, such as ‘indecent’ expression or media materials harmful to juveniles, which may lead to ineffective limits on the actions of these agencies when enforcing the legislation and should not be completely prohibited in a society that recognizes the value of free expression. The Justices emphasized the importance of ensuring that restrictions on communication are clearly defined and do not unduly limit constitutionally protected forms of expression. Imposing limitations on expression based on potential harm that remained uncertain runs counter to fundamental principles of avoiding excessive restriction on individual liberty. Under-regulation is considered preferable to excessive control, emphasizing the importance of allowing a democratic society's inherent self-correcting mechanisms, such as the competition of ideas, to manage value judgments as opposed to legislative regulation that may interfere with freedom of expression.

Article 47(1) of the Electric Telecommunication Act also faced scrutiny in relation to the dissemination of false information and the criteria for 'the intent to harm the public interest, in cases 2008Hun-Ba157 and 2009Hun-Ba88 due to its ambiguity. In 2008Hun-Ba157, the petitioner was charged with violating Article 47(1) of the Electric Telecommunication Act by posting false information on an internet site claiming that a woman was assaulted by police during a protest. Similarly, the petitioner in 2009Hun-Ba88 was charged with the same offense for posting untrue statements on an internet site alleging that currency exchange was halted due to the Korean foreign reserve being drained, and that the Korean government had instructed major Korean banks and export companies to stop buying dollars. Both petitioners filed motions for a constitutional review of the Instant Provision after their petitions were denied by trial courts. The specific issue under scrutiny in these cases is the constitutionality of Article 47(1) of the Electric Telecommunication Act, referred to as the ‘Instant Provision’, which criminalizes individuals who transmit false communication through electronic communication facilities with the intent to harm public interest (22–2(B) KCCR 684, 2008Hun-Ba157, 2009Hun-Ba88(Consolidated), December 28, 2010).

As this provision penalized individuals for the dissemination of false information with an ‘intent to harm the public interest’ (2008Hun-Ba157, 2009Hun-Ba88 2010), the issue under discussion concerned the clarification of the terms as to what exactly amounts to an intent to harm the public interest. The cases focused specifically on the interpretation of public interest, the intent behind the transmission of false information, and potential impacts on fundamental rights.

The court evaluated whether the abstract nature of the concept of public interest in the Instant Provision violates the principle of *nulla poena sine lege* (no punishment without a law) and the rule of clarity. The case raised concerns about the potential for arbitrary enforcement and penalization due to a lack of clear guidance on what exactly constitutes harm to the public interest. The complainants contended that the ambiguity surrounding the concept of public interest could result in restrictions on freedom of expression and undermine the rule of law (Constitutional Court of Korea 2010). The majority opinion is concerned with the constitutionality of the law in relation to freedom of expression and the rule of clarity in criminal laws. They held the view that the specific intent requirement of the ‘intent to harm public interest’ narrowed the scope of punishable acts, reducing the need for a higher level of clarity compared to crimes requiring general intent. ‘Public interest’ was interpreted as the interest of the majority of citizens in Korea and the state. They emphasized that the legislative intent behind the provision was clear in targeting acts with the major intent to harm the public interest. They also highlighted that false information should be interpreted generally in order to cover both false contents and false pretenses, asserting that the term ‘false information’ was sufficiently clear and did not violate the rule of clarity. The majority held the view that the Instant Provision was constitutional, contending that the specific intent requirement and the understanding of public interest provided adequate clarity for enforcement without infringing on freedom of expression. However, in a dissenting opinion, Justice Lee, Dong-Heub, and Justice Mok, Young-Joon, raised concerns about the potential for arbitrary enforcement of the law due to the vagueness of the criteria defining harm to public interest. They believed that the ambiguity surrounding the concept of public interest may lead to limitations on the right to freedom of expression and erode the principles of the rule of law. They held the view that the Instant Provision failed to provide a concrete standard for what may constitute harm to the public interest, potentially leading to subjective interpretations and infringements on freedom of expression, concluding the Instant Provision unconstitutional due to its lack of specificity and clarity and potential for excessive restriction on freedom of expression (Constitutional Court of Korea 2002b). Similarly, 97Hun-Ma108 addressed the tensions that exist between freedom of expression and professional practice, and the protection of public health, recognizing that the protection of public health justifies legal restrictions and limitations on freedom of expression in instances, ‘…deemed necessary for national security, maintenance of public order, or promotion of the public interest.’ (Constitutional Court of Korea 2000).

Within the context of regulating the spread of misinformation and regulating online communications, additional provisions under the Act on Promotion of Information and Communications Network Utilization and Information Protection (APICNUIP), also hold various implications. Article 44–3(1) grants information and communications service providers discretionary authority to take temporary measures when they identify information circulating through their networks intruding on privacy, defaming individuals, or violating their rights.

Moreover, Article 70(2) of the Act addresses defamation via false information that is disseminated across information and communications networks, imposing penalties such as imprisonment with labor, suspension of qualifications, or fines. There does exist, however, a significant regulatory gap in addressing the dissemination of false information that targets an unspecified, and widespread audience with a view to impacting the legal interests of both the nation and society (Hwang and Kwon 2017). The Supreme Court’s decision in 2008Da53812 has also faced criticism in various spheres (Jong 2010). The case assessed whether web portal sites Naver, Daum, SK Communications, and Yahoo Korea could be held liable for defamation. The plaintiff was accused in user postings on these sites of persuading his girlfriend to have an abortion, which led to her suicide after a subsequent pregnancy (Supreme Court en banc Decision 2008Da53812). The court upheld judgments against these services for amounts ranging from 5 million SKW to 10 million SKW. While freedom of the press is a fundamental right, media entities are expected to maintain journalistic integrity and ethical standards by verifying facts, offering balanced reporting, and refraining from spreading misinformation or biased opinions that could potentially damage the reputation of individuals or organizations. The decision 2008Da53812 was concerned with the duty of Internet information service providers to delete or block defamatory posts. The majority opinion held the view that providers have a duty to delete unlawful posts where they are specifically recognized or obviously recognized as defamatory. The minority opinion expressed an alternative perspective and highlighted the importance of freedom of expression in limiting the duty of Internet information service providers to delete content. They contended that the service provider should only be required to remove content if the harmful nature of the content is clear and obvious, implying that a direct request from the affected individual should be necessary for action to be taken. Taking the legal context of the Supreme Court Decision 2008Da53812 into account, media companies are obligated to uphold the accuracy of the information they disseminate and to refrain from propagating falsehoods or distorted opinions. Any intermediaries, including online portals, should share liability for illegal content to the same degree as news agencies subject to the following conditions: the nature of the content’s illegality must be unequivocal, the platform or portal must possess knowledge of the content, and the financial or technical capacity to control the content must exist (Supreme Court of Korea 2009). In addition to an obligation to promptly remove such content, these intermediaries—in this case, content hosting platforms and social media portals are also mandated to prevent similar uploads and posts in the future. The decision establishes a precedent potentially impacting how portals handle misinformation about the pandemic. Portals may be required to actively monitor and remove false information, even without specific complaints or reports from individuals, if the falseness of the information can be easily identified. The ruling implied that revenue-earning portals are expected to proactively monitor and delete such content, blurring the lines between their social responsibilities and legal obligations.

In response to the limitations of current laws addressing the issues posed by misinformation and disinformation, the National Assembly has introduced multiple bills with the aim of regulating and managing these issues more effectively. However, the proposed bills are also perceived to have key issues related to the ambiguous nature of the terminology used (Jung et al. 2021). Unclear definitions of concepts like ‘fake news’ or mis-/disinformation (Jung et al. 2021), the potential for unnecessary or excessive regulation (Jung et al. 2021), and the imposition of excessive or stringent legal liability on information and communication service providers have all been highlighted as potential issues. Laws concerning misinformation have remained inconsistent. Legislated responses to managing social issues are often reactive as opposed to proactive. This is particularly applicable to cases of health misinformation The inherent delay in legal measures, coupled with the challenge of regulating online spaces, especially those offering anonymity, raises questions about the material efficacy of legal measures in response to the problems posed by the spread of health misinformation. Protection of the public welfare has been treated as a legitimate justification for limitations on the individual right to freedom of expression. The theme concerning the issues with existing legislation in South Korea point to the inherent difficulty of defining terms and balancing the welfare of the public with individual freedom of expression and the ongoing effort to deal with the evolving media landscape via legal means. The conceptual ambiguity of legal terms makes regulation challenging in cases where decisions need to be rapid and unequivocal, making the legal wrangling over the scope of specific terms an unnecessary impediment to regulatory action.

### Government Policies as a Response to the COVID-19 Pandemic

The government swiftly implemented various public health measures, such as mask mandates and social distancing protocols, but faced challenges in addressing the concurrent infodemic. Policies targeting the spreading of misinformation, such as amendments to telecommunications laws encountered legal, ethical, and logistical barriers. These included concerns over freedom of expression and the effectiveness of legal measures against COVID-19 misinformation. Governmental strategies evolved to encompass collaborative efforts with private sectors and media outlets, suggesting a recognition of the need for more comprehensive approaches. These strategies reflected efforts of the South Korean government to address the limitations of legislation by actively controlling the spread of misinformation while avoiding excessive restriction of freedom of expression. Several government agencies introduced policies that emphasized flexibility and regulating the spread of information in a context-dependent manner. This is exemplified by the KCC’s introduction of a Vaccine Disinformation Reporting Board specifically targeted at COVID-19 vaccine-related false information. An additional development as part of this response was collaboration between different agencies to combat the spread of COVID-19 misinformation, such as the establishment of a ‘Promotion and Fake News Response Council’ involving six different government agencies. Additionally, as part of the response to health misinformation, KISO implemented the ‘Policy on False and Manipulated Information Regarding COVID-19 in 2021’. Article 34 states the obligation for member companies to identify and address false information related to COVID-19 in cases where the ‘member company becomes aware that a post…is being circulated in relation to COVID-19 information, the company may take necessary actions such as deletion’ (KISO 2021b). This obligation applies to (1) posts containing information about COVID-19 such as its existence, treatment, prevention, diagnosis, infection, social distancing, and self-isolation, and (2) in cases where the information has been clearly confirmed as false by the World Health Organization (WHO) or the Disease Control and Prevention Agency (KISO 2021b). Under the remit of Article 34 Member companies are authorized to take necessary actions, such as post deletion, when dealing with COVID-19-related posts meeting specific criteria, including information on the existence, treatment, prevention, diagnosis, infection, social distancing, and self-isolation. Furthermore, if the information has been confirmed as false by reputable sources like the World Health Organization (WHO) or the Disease Control and Prevention Agency, companies are encouraged to act promptly to prevent the spread of misinformation. KISO was instrumental in enforcing these regulations, with a focus on targeting false claims related to ‘vaccine prevention’, ‘treatment’, ‘existence’, and ‘transmission’. From April to October, KISO undertook actions against 1538 false COVID-19-related posts on social media and online portals, demonstrating a proactive approach to combating misinformation and safeguarding public health (KISO 2021a).

### Self-Regulation in the Private Sector

A further theme emerged in relation to the self-regulation in the private sector and highlights the proactive approach taken by private sector companies in South Korea with the aim of addressing health misinformation during the COVID-19 pandemic. Domestic sites, such as NAVER and Kakao, implemented their own policies targeting COVID-19 misinformation in alignment with government announcements and the introduction of tools to aid in public awareness. Global platforms with substantial presence in South Korea also made individual efforts to combat the dissemination of false information using various methods with Instagram blocking hashtags related to fake news surrounding the COVID-19 vaccine. Prominent media associations in South Korea developed and adhered to internal guidelines centered around reporting during an epidemic illustrating the benefits of a collaborative approach to misinformation management where private sector companies act to complement governmental strategies. These self-regulatory measures aligned with government strategies and constituted an important part of the South Korean response to health misinformation. However, while this theme highlights the importance of blending government intervention with corporate responsibility and public engagement, further investigation is needed to assess the material effectiveness and long-term sustainability of this type of multi-stakeholder approach to tackling health misinformation, especially in the sphere of rapid online communication.

### Mitigation of Social Impacts of COVID-19 Misinformation

This theme illuminates the dualistic nature of the strategy employed by South Korea’s institutions in efforts to mitigate the social impact of COVID-19 misinformation, where the focus of institutions was on both the removal of false information while simultaneously informing and educating the public about the false claims and misinformation.

The internet has democratized access to medical knowledge, but there is the potential for claims to spread unchecked among individuals and communities who may lack the necessary language, context, or education to understand the nuanced nature of particular types of information (Burke 2020). This may lead to misguided health decisions based on misunderstandings or mistrust related to healthcare and further foster skepticism towards government and medical authorities (Lăzăroiu et al. 2021). Early studies have suggested that nearly 6000 people were hospitalized and 800 people died due to COVID-19 misinformation (Islam et al. 2020, as cited by WHO 2021c). Health misinformation, often underpinned by rumors, stigmatization, and conspiracy theories, has potentially severe adverse implications for public health especially in instances where they run parallel to or even overpower correct information and reliable sources of messaging and influence individuals’ healthcare-seeking behaviors, potentially dissuading them from undergoing essential measures such as COVID-19 testing or turning to false or untested cures (Bok et al. 2021).

Approaches to managing the spread of misinformation have involved the removal of false content coordinated between the government agencies and private sector companies but also proactive public education efforts. Many of the government strategies were focused on the former, restricting the spread of misinformation through the active removal of false content. For instance, KISO implemented policy frameworks for the removal of COVID-19-related misinformation and introduced policies authorizing the removal of exclusively false or misleading COVID-19-related content, and the Broadcasting Commission acknowledged having taken corrective measures against COVID-19 information. Concurrently, the ‘Promotion and Fake News Response Council’ developed digital content to promote accurate information in collaboration with government agencies as part of their comprehensive measures incorporating digital content such as card news and video clips to promote correct information. Similar efforts have been made in the private sector. Anti-vaccine channels have been blocked on Youtube, and articles containing false information have been flagged on Facebook. While the measures taken were specific to each individual platform, they are similar in that they aim to contain misinformation and prevent their further dissemination. There have been efforts in both the governmental and private sectors to inform/educate the public on correct information. While the removal of misinformation is a direct response, the success of public education initiatives in changing public perceptions remains an area for further investigation as to the nature of their effectiveness and impact on public trust in health communication.

**Fig. 1:**
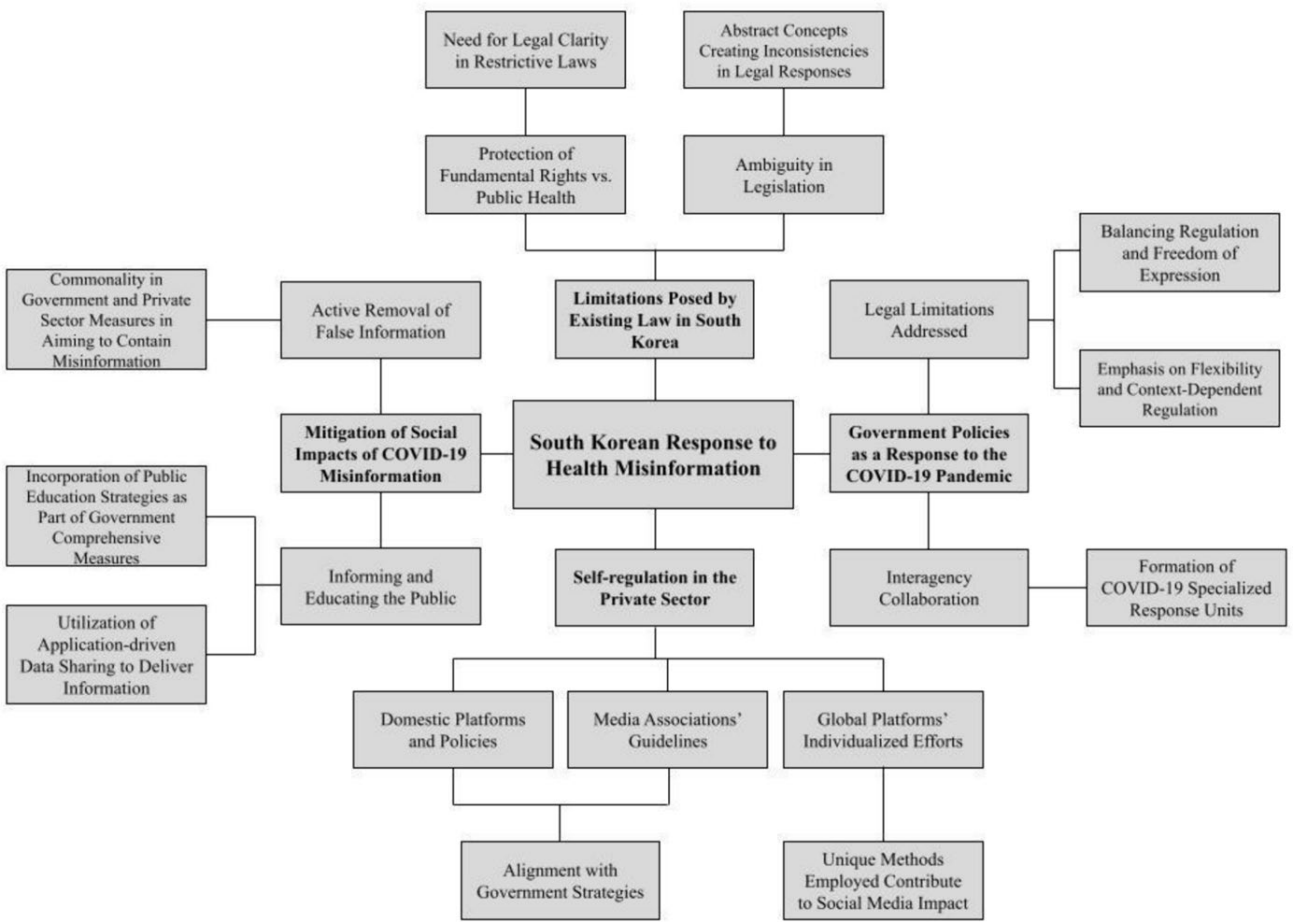
Themes and sub-themes identified following thematic analysis of South Korea’s response to health misinformation

Figure 1 illustrates the course of theme identification undertaken throughout the literature review.

## Limitations

While efforts have been made to keep the paper comprehensive in scope, certain limitations have been faced in its conception and execution. Firstly, the reliance on publicly available documents such as press releases issued by governmental institutions potentially erases the multidimensional nature of policy development and limits perspectives to the institutions themselves, especially in the absence of robust data sources. Secondly, the fact that health misinformation remains an evolving issue means that some of the conclusions may be outdated at the time of submission and that later developments remain unaccounted for. Additionally, while this paper focuses on the South Korean perspective, difficulties were faced with translation, and the selection of appropriate terminology meaning that some of the nuance in legal terminology and critique may have been lost in translation, although consultation with native speakers aimed to minimize this potential issue. Similarly, conclusions, while useful for considering the South Korean context, may lack universality. In terms of the type of study undertaken, the thematic analysis approach, while effective for broadly identifying and analyzing key themes, has the potential to miss more nuanced insights owing to the way that data was categorized. Finally, this paper is primarily concerned with the legal and policy dimensions of managing health misinformation which does not accommodate wider impact factors such as psychological or community aspects.

## Conclusion

The pandemic has highlighted the importance of curbing the spread of misinformation and making sure that people have easy access to clear, up-to-date, and correct information, but there are also long-term implications associated with self-regulation, as seen by government organizations like KISO as well as self-regulation initiatives from companies operating in the private sector, as policy measures require ongoing adaptation and refinement in order to effectively address evolving challenges associated with health misinformation management. South Korea’s collaborative multi-stakeholder approach, identified through this narrative-driven thematic analysis, offers a useful perspective on South Korea’s policy response to health misinformation during the COVID-19 pandemic. The themes identified address the question of how effectively South Korea’s policy interventions and legal measures managed health misinformation challenges. However, there are also opportunities for future investigation owing to the recent nature and lack of robust data on the nature of stakeholder responses to infodemics. This investigation offers unique perspectives into the role of multi-stakeholder collaboration in managing health misinformation, with an emphasis on the role of comprehensive policy development, public–private partnerships, and public health education and digital literacy in order to address the evolving nature of infodemics.

The key themes such as legal responses, government strategies, private sector self-regulation, and public education efforts demonstrate the inherent complexity and dynamic nature of infodemic management. While South Korea’s integrated approach has shown initiative in many respects, the rapidly evolving nature of online spaces is likely to pose ongoing and future challenges, signaling a need for ongoing adaptation, evaluation, and innovation of policy and legislative measures. Investigating the long-term impact of these strategies, especially in terms of public trust and behavioral change or response, is crucial to understanding the most effective methods for managing the spread of misinformation. Exploring the efficacy of public–private partnerships in real-time misinformation management is another avenue for investigation.

Self-regulation by private companies emerged as a crucial area for consideration as to the nature of their long-term viability. The dual approach of removing misinformation and promoting accurate information emphasized the importance of informed public engagement in the fight against misinformation. Approaches to addressing mis-/disinformation vary greatly both internationally and domestically and the sheer volume of information circulating on the internet and social media platforms makes it logistically difficult to monitor and prosecute every case of misinformation dissemination. Misinformation is still a growing area of study in terms of knowing the extent of its effects on public and global health and remains contested in academic circles (Vraga and Bode 2020). The impacts of misinformation responses on public health initiatives, including vaccine uptake and public compliance with health guidelines, remain areas largely underexplored throughout the current scholarship. Consequently, there are also opportunities for further research and investigation into the area in the future with a view to identifying specific effects and generating more robust data analyzing the relationships between health misinformation and the success of public health initiatives. Empirical studies may be able to provide additional insight into the efficacy of different misinformation countermeasures. Further research in this area would aid a more comprehensive understanding of the issues and inform the development of more effective public health strategies and policies.

Additionally, the role of trans-border cooperation networks in global infodemic management warrants further consideration as the global nature of the internet means that the issues associated with health misinformation are not contained by borders. In cases of future and emerging public health threats, international collaboration will be vital.

Sharing strategies, data, and resources across borders may aid in the enhancement of the global response to future infodemics, leveraging collective expertise and technology. Adding provisions to international guidance such as International Health Regulations, on managing infodemics for example, could allow nations to coordinate a more cohesive and efficient response to more effectively target the spread of misinformation. There is an opportunity to expand the scope of data monitoring, by the WHO. Expanding WHO’s data monitoring initiatives and capabilities may be able to give crucial insights into the prevalence, trends, and implications of health misinformation on a global scale. The COVID-19 pandemic has demonstrated that a coordinated international response is crucial in terms of not only international organizations such as the WHO or OECD but across private sector companies involved with information communication at the global level. Developing robust transborder networks for information exchange and joint initiatives has the potential to enhance the effectiveness of infodemic management strategies, harmonize legal measures, and foster unified approaches to public health communication. While South Korea’s response to the COVID-19 pandemic has been largely effective and widely praised, the need for continuous innovation and global collaboration in the realm of infodemic management remains clear. Future research should focus on the sustainability of current strategies, the development of new technology and platforms for misinformation management, and the strengthening of international cooperation frameworks.

## Abbreviations

JAK: Journalists Association of Korea
KBJA: Korean Broadcasting Journalist Association
KCC: Korea Communications Commission
KDCA: Korea Disease Control and Prevention Agency
KFDA: Korea Food and Drug Administration
KISO: Korea Internet Self-Governance Organization
KNPA: National Police Agency
KSJA: Korean Science Journalists Association
MCST: Ministry of Culture, Sports and Tourism
MOHW: Ministry of Health and Welfare
